# Hsp90 Relieves Heat Stress-Induced Damage in Mouse Kidneys: Involvement of Antiapoptotic PKM2-AKT and Autophagic HIF-1α Signaling

**DOI:** 10.3390/ijms21051646

**Published:** 2020-02-28

**Authors:** Bixia Chen, Bo Yang, Jie Zhu, Jiaxin Wu, Junzhou Sha, Jiarui Sun, Endong Bao, Xiaohui Zhang

**Affiliations:** Department of Basic Veterinary Medicine, College of Veterinary Medicine, Nanjing Agricultural University, Nanjing 210095, China; 2019807112@njau.edu.cn (B.C.); 2019107002@njau.edu.cn (B.Y.); 2019107001@njau.edu.cn (J.Z.); 2018807111@njau.edu.cn (J.W.); 2018107002@njau.edu.cn (J.S.); 2016207004@njau.edu.cn (J.S.); b_endong@njau.edu.cn (E.B.)

**Keywords:** heat stress, kidney, Hsp90, apoptosis and autophagy, signal pathway

## Abstract

Heat stress can particularly affect the kidney because of its high rate of adenosine triphosphate consumption. Competition between apoptosis and autophagy-mediated survival always exists in damaged tissue. And Hsp90 can enhance cellular protection to resist heat stress. However, the relationship between Hsp90 and the above competition and its underlying mechanism in the kidney are unclear. The present study found that heat stress induced obvious histopathological and oxidative injury, which was connected with cellular apoptosis and autophagy in the kidney and was associated with the levels of Hsp90 expression or function. The data showed that during heat stress, Hsp90 activated the PKM2-Akt signaling pathway to exert antiapoptotic effects and induce Hsp70 expression regulated by HSF-1, stimulated autophagy-mediated survival through the HIF-1α-BNIP3/BNIP3L pathway, and finally protected the kidney from heat-stress injury. Moreover, the nuclear translocation of PKM2, (p-) Akt, HSF-1, and HIF-1α was enhanced by heat stress, but only intranuclear p-Akt and HSF-1 were specifically influenced by Hsp90, contributing to regulate the cellular ability of resisting heat-stress damage. Our study provided new insights regarding the molecular mechanism of Hsp90 in the kidney in response to heat-stress injury, possibly contributing to finding new targets for the pharmacological regulation of human or animal acute kidney injury from heat stress in future research.

## 1. Introduction

Heat stress (HS) affects not only humans, such as military recruits and marathon runners, but also livestock (poultry and dairy cattle) and is a common medical problem associated with severe heat exposure that can result in multiorgan failure and even lead to sudden death, with the kidney being particularly affected [1,2,3]. This injury is likely to become more serious with climate change and increasing heat extremes [4,5,6]. Acute stress decreases renal blood flow and results in ischemia, thus inducing acute kidney injury (AKI) [7,8]. The underlying pathogenesis involves injury to nephron segments, both from the ischemia itself and from the mechanism of survival or death in response to oxidative stress [9,10]. Proximal renal tubular cells along the nephron segments are particularly sensitive to hypoxia because of their high rates of adenosine triphosphate consumption [9,10].

Stressful stimuli generates potentially harmful ROS above a physiological threshold, and apoptosis is often a consequence of reactive oxygen species (ROS) generation, triggering cell loss and inducing tissue injury [11]. Several types of stimuli can cause apoptosis and autophagy in the same cell. Autophagy, a lysosomal degradation pathway, is an essential cellular adaptation mechanism for avoiding oxidative damage. Studies of the role of autophagy in AKI have reported both beneficial and detrimental effects [12,13,14]. Light chain 3 (LC3), is essential for autophagy and serves as a vital component in monitoring autophagy and visualizing autophagosomes in vivo [15]. Pyruvate kinase M2 isoform (PKM2), a rate-limiting terminal glycolytic enzyme, catalyzes the last step of glycolysis, and recent studies have reported that PKM2 is associated with the Akt signal pathway, which regulates cell survival and apoptosis [16,17]. Meanwhile, many cellular signals have been reported to be involved in the regulation of autophagy, including the Akt (protein kinase B)-mTOR (mammalian target of rapamycin) pathway [18,19]. Furthermore, Akt also plays a vital role in resisting heat stress-induced apoptosis [20]. Hypoxia-inducible factor (HIF), especially HIF-1α, is also a major actor in the cell survival autophagy in response to hypoxia stress via BNIP3 (Bcl-2/adenovirus E1B 19-kDa interacting protein 3) and BNIP3L (Bcl-2/adenovirus E1B 19-kDa interacting protein 3 like) [21]. However, understanding the roles and their interplay of autophagy and apoptosis, and identification of the closely related signaling pathways in the kidney, is still very deficient but important for controlling tissue damage during heat stress.

At the cellular level, tolerance to heat stress is mainly regulated by heat shock proteins (HSPs), which are also synthesized under various environmental and oxidative stresses in the kidney [22,23,24]. The HSPs that have been most studied in the kidney are 90-kDa (Hsp90) and 70-kDa (Hsp70) [24]. In particular, Hsp90 is a highly-conserved and abundant protein, constituting up to 2% of total cellular proteins that can increase to 6% in stressed conditions [25]. As a molecular chaperone, Hsp90 utilizes its ATPase activity to drive conformational changes to facilitate client protein binding, stabilization, and activation [25,26,27,28]. Intracellular HSPs can function to inhibit apoptosis and facilitate cell proliferation, preserving renal tubule viability after acute injury [24,29]. Although PKM2, Akt, and HIF-1α are well-known client proteins of Hsp90 in kinds of cancer cells [30,31,32], little is known about the role of Hsp90 in autophagy and its relationship with apoptosis in heat stress-induced AKI pathogenesis.

Our previous study in myocardial cells showed that aspirin (ASA), a nonsteroidal antipyretic drug, increased Hsp90 expression, contributing to the resistance to heat stress-induced apoptosis, and that geldanamycin (GA) treatment effectively inhibited its function as a molecular chaperone [20]. In this in vivo study, mice were treated with ASA, GA, and TR (triciribine, the inhibitor of Akt activation) without or with heat stress, for evaluating our hypothesis that heat stress-induced acute kidney injury originated from peroxidation, and that Hsp90 enhanced cellular protection by restricting apoptosis and inducing autophagy-mediated survival by two separate signaling pathways, Hsp90-PKM2-Akt and Hsp90-HIF-1α-BNIP3/BNIP3L.

## 2. Results

### 2.1. Heat-Stressed Kidney Damage under Different Treatments

**Figure 1:**
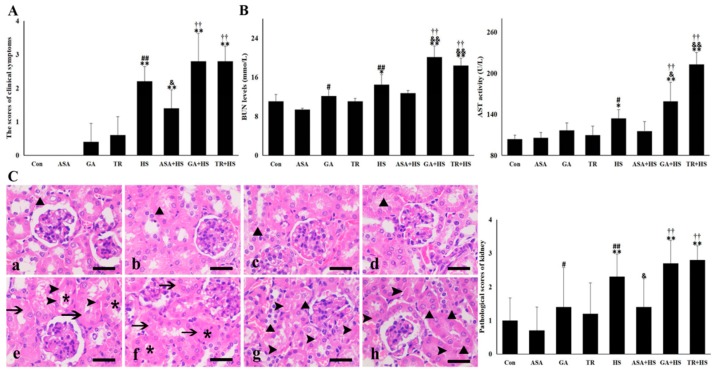
Kidney damages in the different groups. The data represent the means ± SD. (**A**) Clinical symptom scores in the different groups. (**B**) Serum BUN (blood urea nitrogen) and AST (alanine aminotransferase) levels in the different groups. (**C**) Histopathological observations were conducted under a light microscope. Left: a–h show the morphology of the tested renal tissues in the Con, ASA, GA, TR, HS, ASA+HS, GA+HS, and TR+HS groups, respectively, after hematoxylin eosin (H&E) staining. Bar = 20 µm. Arrows indicate swollen cells, asterisks indicate lumen stenosis or atresia, arrowheads mark necrosis, and black triangles show cellular degeneration. Right: Pathological scores of the different groups. The comparison between the Con group and other groups is indicated by * *p* < 0.05 and ** *p* < 0.01, comparison of the ASA group with the GA, TR, and HS groups is indicated by ^#^ *p* < 0.05 and ^##^ *P* < 0.01, comparison of the HS group with the ASA+HS, GA+HS, and TR+HS groups is indicated by ^&^ *p* < 0.05 and ^&&^ *p* < 0.01, and comparison of the ASA+HS group with the GA+HS and TR+HS groups is indicated by ^†^ *p* < 0.05 and ^††^ *p* < 0.01.

Experimental mice in the Con and ASA groups were in good physical condition and without any abnormities in their mental state during the experiment. Several mice treated with GA and TR showed slight mental depression. The animals exposed to HS alone showed increased respiratory rates and uneasiness immediately and then laid down with glazed eyes. Mice in the ASA+HS group moved normally with a slight mental depression and sometimes curled up under the cushion. Animals in the GA+HS and TR+HS groups showed more obvious mental depression most of the time, with increased respiratory rates, and sometimes struggled to free themselves. One mouse in the GA+HS group died during exposure to heat stress. As shown in Figure 1A, the symptom scores of the HS, ASA+HS, GA+HS, and TR+HS groups but not the ASA, GA, and TR groups showed significant (*p* < 0.01) increases compared to those of the Con group. The score of the ASA+HS group was lower (*p* < 0.05) compared to that of the HS group, while those of the GA+HS and TR+HS groups were slightly higher.

Serum enzymes related to renal injury, alanine aminotransferase (AST), and blood urea nitrogen (BUN) were detected. The data in Figure 1B show that heat stress significantly increased the levels of AST and BUN in mouse serum, and GA and TR treatment further enhanced these increases. In particular, the activities of AST and BUN in the ASA+HS group did not show obvious differences from those in the Con group. These data suggest that heat stress caused obvious tissue damage, which was alleviated by ASA administration and enhanced with GA or TR treatment. Histopathological data in Figure 1C show mild hydropic degeneration and granular degeneration in some kidneys in the Con, ASA, GA, and TR groups. In the HS group, epithelial cells in the renal tubules were swollen, contributing to lumen closure, and some were even necrotic and shed into the tubular lumen. Closed lumen and swollen epithelial cells were observed in the ASA+HS group without obvious cell necrosis. For the GA+HS and TR+HS groups, most epithelial cells showed degeneration of different degrees, and more necrotic cells were observed than in the HS group. The pathological scores of the ASA, GA, and TR treatment groups did not adversely influence kidney tissues. However, heat stress evidently increased the damage score of the kidney, which was reversed by ASA treatment and further strengthened by GA and TR administration.

### 2.2. The Oxidative and Antioxidant Condition of the Kidney under Different Treatments

**Figure 2:**
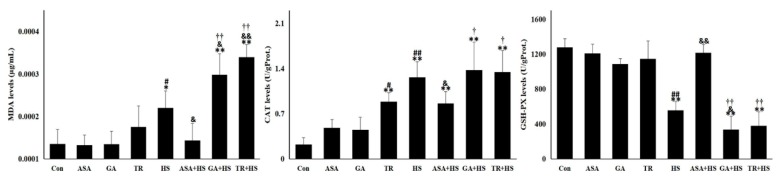
The oxidative and antioxidant conditions in the kidney under different treatments. The levels of MDA (malondialdehyde), CAT (catalase), and GSH-PX (glutathione peroxidase) in the tested kidney tissues of the different groups. Data represent the means ± SD. The comparison between the Con group and other groups is indicated by * *p* < 0.05 and ** *p* < 0.01, comparison of the ASA group with the GA, TR, and HS groups is indicated by ^#^ *p* < 0.05 and ^##^ *p* < 0.01, comparison of the HS group with the ASA+HS, GA+HS, and TR+HS groups is indicated by ^&^ *p* < 0.05 and ^&&^ *p* < 0.01, and comparison of the ASA+HS group with the GA+HS and TR+HS groups is indicated by ^†^ *p* < 0.05 and ^††^ *p* < 0.01.

As shown in Figure 2, renal malondialdehyde (MDA), catalase (CAT), and glutathione peroxidase (GSH-PX) activity levels were unaffected by ASA, GA, and TR treatment, except for an increase in CAT in the TR group. Renal MDA and CAT activity levels increased significantly (*p* < 0.05 or *p* < 0.01) after heat stress treatment, while GSH-PX levels decreased (*p* < 0.01). ASA treatment effectively reversed the HS-induced increase in MDA and CAT activities and the reduction in GSH-PX levels. The MDA levels in the heat-stressed kidney were higher due to GA or TR pretreatment compared to that of the HS group. In addition, GSH-PX activities in the GA+HS and TR+HS groups were lower than those in the HS group, especially the GA+HS group (*p* < 0.05). These data suggest that heat stress results in serious oxidative damage, which was relieved by ASA administration and enhanced with GA or TR treatment.

### 2.3. Hsp90 Correlates with Cellular Apoptosis and Autophagy-Mediated Survival in Conjunction with Hsp70

**Figure 3:**
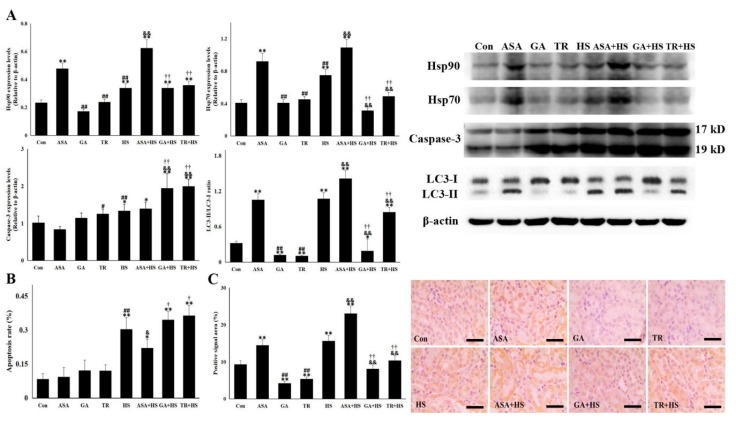
Hsp90 and Hsp70 are related to cellular apoptosis and autophagy-mediated survival. The data represent the means ± SD. (**A**) Total proteins of the tested kidney tissues were used for Western blot detection of the corresponding proteins. The relative abundance of the tested proteins was normalized to that of β-actin. (**B**) Paraffin-embedded tissues were sliced for the detection of apoptotic cells by TUNEL (TdT-Mediated dUTP Nick End Labeling) staining. Ratio of apoptotic cells to normal cells determined the apoptosis rate. (**C**) Immunohistochemistry staining was performed using an anti-LC3-II antibody. The positive signal areas (%) of the stained sections were quantified using Image J software (Left). Representative images from the Con, ASA, GA, TR, HS, ASA+HS, GA+HS, and TR+HS groups are presented (Right). Bar = 20 µm. The comparison between the Con group and other groups is indicated by * *p* < 0.05 and ** *p* < 0.01, comparison of the ASA group with the GA, TR, and HS groups is indicated by # *p* < 0.05 and ^##^ *p* < 0.01, comparison of the HS group with the ASA+HS, GA+HS, and TR+HS groups is indicated by ^&^ *p* < 0.05 and ^&&^ *p* < 0.01, and comparison of the ASA+HS groups with the GA+HS and TR+HS groups is indicated by ^†^ *p* < 0.05 and ^††^ *p* < 0.01.

Next, we detected the levels of two vital heat shock proteins, Hsp90 and Hsp70, in the kidney. The results in Figure 3A show that heat stress stimulated the expression of Hsp90 and Hsp70, and ASA further induced expression of both proteins with or without exposure to heat stress. Despite no influence in tissues without heat stress, GA or TR treatment significantly restricted renal Hsp70 levels in heat-stressed mice and did not alter Hsp90 levels compared to those in the HS group.

Figure 3A,B shows that ASA did not influence apoptosis-related Caspase-3 levels in kidneys exposed to heat stress, while the heat stress-induced apoptosis rate of renal tubular epithelial cells in ASA-treated tissue was significantly lower than that in kidneys exposed to heat stress alone. GA or TR alone increased Caspase-3 levels, which were further significantly strengthened by heat stress, which was in agreement with the apoptosis rate in the corresponding groups. Whether autophagy was altered by the different treatments was also investigated (Figure 3A). Western blotting showed that heat stress induced an apparent increase in LC3-II in renal tissues, which was further strengthened by ASA treatment, even in tissues that were not heat stressed. However, after GA or TR administration, the LC3-II levels were markedly decreased in kidneys, with or without heat stress. This observation was also verified by the similar results of positive signal areas in immunohistochemical staining (Figure 3C). These data demonstrate that the inhibition of apoptosis and the occurrence of autophagy-mediated survival in heat-stressed kidneys was closely associated with the expression or function of Hsp90, which might be assisted by Hsp70 [30]. Moreover, the deficiency in Akt activation with TR treatment may restrict the autophagy level, contrary to a previous study [33].

### 2.4. The Antiapoptotic Pathway Hsp90-PKM2-Akt Signaling Is Affected by Different Treatments

**Figure 4:**
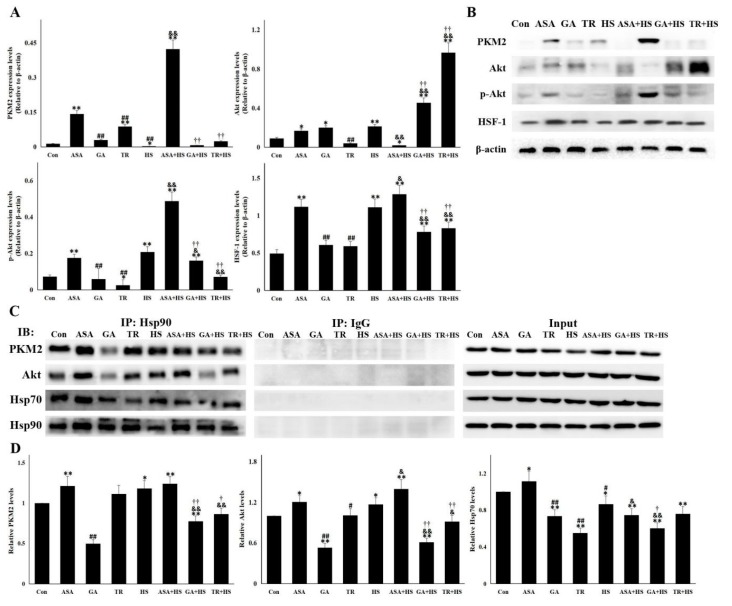
PKM2-Akt signaling was activated to inhibit apoptosis through its interaction with Hsp90. Data represent the means ± SD. (**A**,**B**) Total proteins of tested kidney tissues were used for Western blot detection of the corresponding proteins. (**A**) Reactive bands were quantified using Quantity One software, and the relative abundance of the tested proteins was normalized to that of β-actin. (**B**) Representative bands are presented. (**C**,**D**) Co-immunoprecipitation was conducted to investigate the interaction of Hsp90 with PKM2, Akt, and Hsp70. (**C**) Representative bands are presented. (**D**) The reactive bands from co-immunoprecipitation detection were quantified using Image J software. Intensities of bands in Con were set as 1. The comparison between the Con group and other groups is indicated by * *p* < 0.05 and ** *p* < 0.01, comparison of the ASA group with the GA, TR, and HS groups is indicated by # *p* < 0.05 and ^##^ *p* < 0.01, comparison of the HS group with the ASA+HS, GA+HS, and TR+HS groups is indicated by ^&^ *p* < 0.05 and ^&&^ *p* < 0.01, and comparison of the ASA+HS group with the GA+HS and TR+HS groups is indicated by ^†^ *p* < 0.05 and ^††^ *p* < 0.01.

It was recently identified that the Hsp90 target protein PKM2 was pivotal for inhibiting cellular apoptosis [31,34]. PKM2 and its downstream targets were detected in this study (Figure 4A,B). Contrary to the apoptosis levels in each group, PKM2 expression was seriously restricted in the HS group but was evidently induced by ASA, with or without heat stress. Although the upregulation of PKM2 was observed to different degrees in the GA and TR groups, PKM2 remained at rather low levels in the GA+HS and TR+HS groups. After exposure to HS, the induction of Akt and its activation were observed and further strengthened by the effect of ASA. Upon treatment with GA or TR, heat stress did not stimulate p-Akt production in the GA+HS and TR+HS groups, resulting in the accumulation of Akt protein. p-Akt levels in the GA+HS and TR+HS groups were significantly lower than those in the HS and ASA+HS groups. As a downstream Akt signal and regulator of HSP expression, HSF-1 expression in each group was similar to that of p-Akt. Hsp90 functions on its target proteins through its interaction, which was analyzed by using immunoprecipitation (Figure 4C,D). The results showed that heat stress induced more interaction of Hsp90 with PKM2 and Akt, which was strengthened by ASA administration, regardless of heat stress. When GA inhibited the chaperone function of Hsp90, its interactions with PKM2 and Akt in the GA and GA+HS groups was restricted to different degrees. TR treatment did not obviously disturb the interaction of Hsp90 with these target proteins, regardless of heat stress. Hsp70 promotes the chaperone function of Hsp90 through its interaction with the Hsp90 complex [30]. Immunoprecipitation showed that heat stress slightly downregulated the interaction between Hsp90 and Hsp70. However, ASA strengthened this interaction, which was slightly weakened after exposure to heat stress. GA or TR treatment resulted in obvious restriction of this interaction, which continued during heat stress.

### 2.5. The Nuclear PKM2, Akt, p-Akt, and HSF-1 Is Also Altered for Their Renal Protection

**Figure 5:**
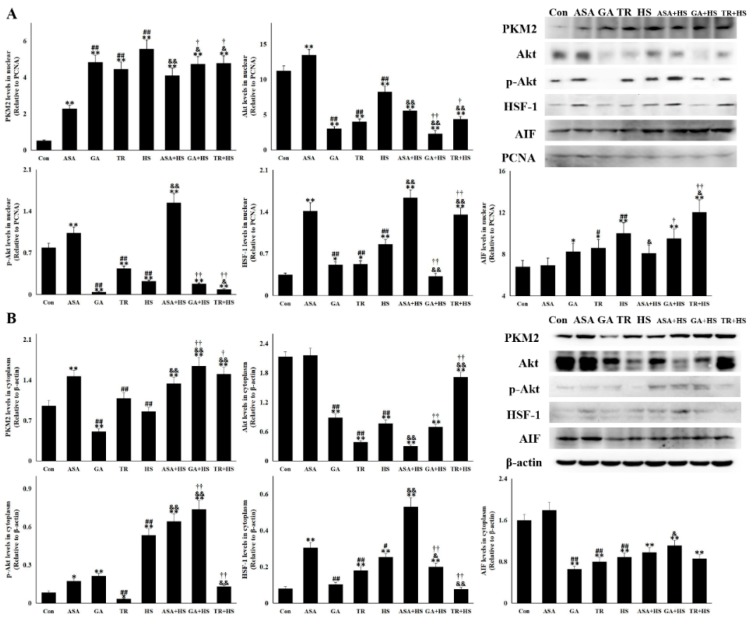
The nuclear and cytoplasmic levels of vital proteins in the different groups. Western blot analysis was performed with the indicated antibodies. The data represent the means ± SD. (**A**) Nuclear proteins of renal tissues in all tested groups were extracted and used for semiquantitative detection of the corresponding proteins. The relative abundance of the tested proteins was normalized to that of PCNA (proliferating cell nuclear antigen). (**B**) Cytoplasmic proteins of renal tissues in all tested groups were extracted and used for semiquantitative detection of the corresponding proteins. The relative abundance of the tested proteins was normalized to that of β-actin. The comparison between the Con group and other groups is indicated by * *p* < 0.05 and ** *p* < 0.01, comparison of the ASA group with the GA, TR, and HS groups is indicated by # *p* < 0.05 and ^##^ *p* < 0.01, comparison of the HS group with the ASA+HS, GA+HS, and TR+HS groups is indicated by ^&^ *p* < 0.05 and ^&&^ *p* < 0.01, and comparison of the ASA+HS group with the GA+HS and TR+HS groups is indicated by ^†^ *p* < 0.05 and ^††^ *p* < 0.01.

Nuclear translocation of PKM2 is instrumental in promoting cell proliferation and development, and p-Akt translocation into the nucleus plays an important role in inhibiting cellular apoptosis [11,35]. Detection of renal nuclear proteins in Figure 5A shows that heat stress induced the most PKM2 translocation into the nucleus, followed by GA, TR, GA+HS, and TR+HS. ASA also stimulated PKM2 nuclear translocation, which was strengthened after heat stress and was lower than those in the GA+HS and TR+HS groups. Heat stress significantly intensified the consumption of the nuclear Akt and p-Akt, which was further enhanced by GA or TR, regardless of heat stress. ASA treatment promoted Akt and p-Akt accumulation in the nucleus, especially p-Akt in the heat-stressed kidney. Upregulation of nuclear HSF-1 caused by heat stress was further stimulated by ASA, while GA or TR differentially inhibited this induction. Apoptosis-inducing factor (AIF) is an important indicator of apoptosis in the nucleus. Western blotting showed that nuclear AIF in the HS group was increased, while ASA effectively restricted this rise, and GA/TR also/further increased its upregulation.

The cytoplasmic proteins are shown in Figure 5B, and the PKM2 level was not increased by heat stress but was increased in the ASA, ASA+HS, GA+HS, and TR+HS groups. Akt was decreased in the different groups to various degrees, except for the ASA group. p-Akt was induced in the HS, ASA, ASA+HS, GA, and GA+HS groups. TR significantly decreased the cytoplasmic p-Akt levels, regardless of heat stress. The cytoplasmic HSF-1 level was enhanced by heat stress and further induced by ASA plus HS, but was inhibited in the GA+HS and TR+HS groups. Cytoplasmic AIF (Apoptosis inducing factor) in all treated groups was downregulated differently, except for the ASA group.

### 2.6. The Hsp90-HIF-1α-BNIP3/BNIP3L Pathway Was Correlated with Heat Stress-Mediated Autophagy

**Figure 6:**
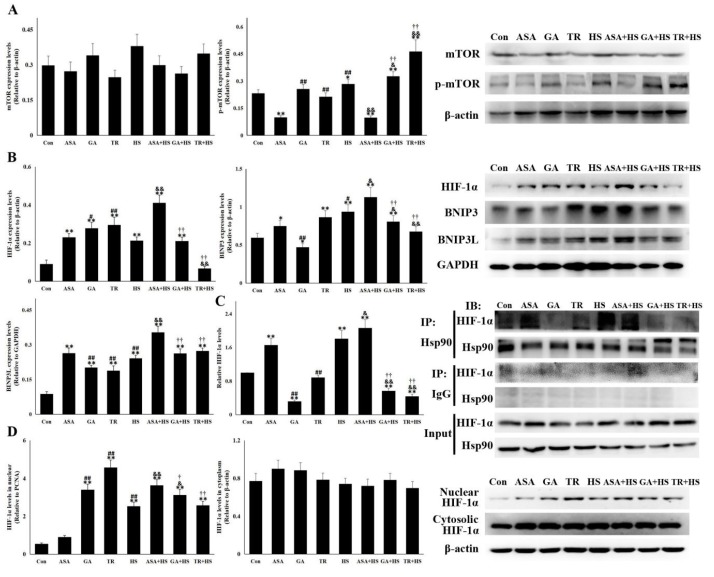
The HIF-1α-BNIP3/BNIP3L pathway is involved in heat stress-induced autophagy. With the indicated antibodies, Western blot analysis was used to detect the levels of different proteins, and coimmunoprecipitation was used to detect the interaction of Hsp90 with HIF-1α. Data represent the means ± SD. (**A**) Total proteins of tested kidney tissues from all groups were analyzed for the levels of mTOR and p-mTOR by Western blotting. The relative abundance of the tested proteins was normalized to that of β-actin. (**B**) Total proteins of tested kidney tissues from all groups were used to detect the levels of HIF-1α, BNIP3, and BNIP3L by Western blotting. The relative abundance of the tested proteins was normalized to that of β-actin. (**C**) Total proteins of tested kidney tissues from all groups were subjected to coimmunoprecipitation to observe the interaction of Hsp90 with HIF-1α. The reactive bands from co-immunoprecipitation analysis were quantified using Image J software (Left). The abundances of bands in Con were set as 1. Representative bands are presented (Right). (**D**) Nuclear and cytoplasmic proteins were extracted to detect HIF-1α levels. The relative abundance of the tested proteins was normalized to that of PCNA (band not shown) and β-actin. The comparison between the Con group and other groups is indicated by * *p* < 0.05 and ** *p* < 0.01, comparison of the ASA group with the GA, TR, and HS groups is indicated by # *P* < 0.05 and ^##^ *p* < 0.01, comparison of the HS group with the ASA+HS, GA+HS, and TR+HS groups is indicated by ^&^ *p* < 0.05 and ^&&^ *p* < 0.01, and comparison of the ASA+HS group with the GA+HS and TR+HS groups is indicated by ^†^ *p* < 0.05 and ^††^ *p* < 0.01.

Pathways related to autophagy-mediate survival in heat-stressed kidneys were also analyzed in this study. As a downstream target of Akt, mTOR and its phosphorylation were first detected (Figure 6A). mTOR levels were not obviously altered by the different treatments. However, an increase in p-mTOR was observed in HS and was enhanced by GA or TR administration. Unexpectedly, p-mTOR was restricted significantly in the ASA and ASA+HS groups, which was contrary to Akt activation. Increasing evidence suggests that HIF-1α plays an important role in autophagy progression by either repressing the mTOR complex or enhancing BNIP3/BNIP3L, which recovers autophagic function of Beclin-1 [21,36,37]. HIF-1α, BNIP3 and BNIP3L expression was investigated (Figure 6B). Renal HIF-1α showed an obvious induction in all experimental groups except for the TR+HS group, especially in the ASA+HS group. Western blotting also showed that heat stress significantly stimulated (*p* < 0.05) BNIP3 and BNIP3L expression, which were further enhanced by ASA treatment, regardless of heat stress. Although GA or TR alone upregulated renal BNIP3 and BNIP3L levels, their induction in the GA+HS and TR+HS groups was markedly restricted by heat stress, especially BNIP3, compared to that of the HS group. The interaction between HIF-1α and Hsp90 was analyzed by coimmunoprecipitation (Figure 6C). The interaction was enhanced by heat stress alone and ASA, with or without heat stress. GA alone but not TR obviously inhibited the chaperone function of Hsp90, while the complex of Hsp90 and HIF-1α in the GA+HS and TR+HS groups was consumed heavily or was not supplemented during heat stress. Nuclear translocation shown in Figure 6D indicates that heat stress, including GA or TR alone, significantly stimulated the accumulation of HIF-1α in the nucleus. After exposure to heat stress, the nuclear HIF-1α level in the ASA+HS group was the highest compared to those in the HS, GA+HS, and TR+HS groups. Cytoplasmic HIF-1α did not display apparent alterations in all experimental groups.

## 3. Discussion

Previous reports have shown that heat stress results in an imbalance between the generation of ROS and the activity of the antioxidant system, causing cellular changes such as lipid peroxidation, DNA damage, and ultimately cell injury [38]. These findings are in agreement with our detection of significant upregulation of renal MDA and CAT, including a decrease in GSH-PX, in the HS group. As expected, obvious kidney injury caused by heat stress was also observed in the HS group, characterized by the increase in AKI indicators (levels of related enzymes and histopathological scores). Wherein, serum AST is increasingly used to estimate the renal injury in the nephritis [39]. In our present study, the indicative role of AST in renal injury was further verified in heat-stressed kidney tissues, in consideration of its consistency with BUN pattern. Therefore, this study supplies a new indicator for detecting the severity of renal injury under the condition of heat stress.

HSPs regulated by HSF-1 are considered central players in the resistance of immature kidneys to ischemic or anoxic injury, especially at the cellular level in renal tubules [24]. More Hsp90 and Hsp70 were synthesized in heat-stressed kidneys, which was accompanied by increases in apoptosis and autophagy. This suggests that the expression of the two mentioned HSPs is closely related, but insufficient to maintain intracellular homeostasis. To this extent, some studies have reported that, in responses to oxidative stress, inducing HSPs with physiological stimulus such as exercise can improve tissue protection [40,41,42]. In this study, aspirin, a well-known anti-fever drug, was proved to induce successfully increased Hsp90 and Hsp70 expression in kidneys with or without heat stress exposure, similar to our studies in myocardial cells [43,44]. The induction of Hsp90 and Hsp70 by aspirin might occur through regulating the expression and nuclear translocation of HSF-1 and HSF-3 [30,43,44]. Under this condition, the inhibition of cellular apoptosis and enhancement of autophagy were found, including milder tissue injury.

This study then focused on how Hsp90 regulated renal cellular apoptosis and autophagy. The interaction of Hsp90 with PKM2 and Akt has been found in oxidative stress and tumor experiments [30,31]. PKM2 serves as an upstream molecule of Akt to regulate Akt expression and the phosphorylation of Akt-1 substrate 1 [33,45]. Consistently, heat stress stimulated the functional consumption of PKM2 to maintain Akt protein levels and enhance its activation through the sustained interaction of Hsp90 and PKM2. Furthermore, the positive effect of Akt on HSF-1 expression also benefited from heat stress-induced reinforcement of the interaction between Hsp90 and Akt. Phosphorylated Akt promotes the cell survival pathway by inactivating certain pro-apoptotic mediators, such as transcription factors of the forkhead (FOXO) family or the Bcl-2 antagonist Bad, and activating Bcl-2 to maintain mitochondrial structure [11,46,47,48]. In multiple myeloma cells, the Akt signaling pathway also stabilizes the expression of HSF-1, thereby controlling constitutive and inducible expression of Hsp70, which is critically dependent on Hsp90 chaperone function [30]. In heat-stressed kidneys, Hsp90 also stimulated Hsp70 synthesis through the PKM2-Akt-HSF-1 axis, and higher expression of Hsp90 from aspirin treatment further intensified this stimulation. Moreover, during heat stress, the binding of Hsp70 with the Hsp90 complex became more transient, and faster turnover may be helpful for triggering more downstream survival proteins.

PKM2 is expressed in all proliferating cells that commonly present high rates of nucleic acid synthesis, such as embryonic cells, adult stem cells, and especially tumor cells [49]. PKM2 translocates into the nucleus to modulate the actions of transcription factors in different cell types [50,51,52]. In addition, p-Akt and its presence in the nucleus can inhibit increases in Foxo3α levels and its transport into the nucleus. Consequently, Foxo3α-dependent transcription of the apoptotic target genes p27, Bim, and Fas-L is effectively restricted, inhibiting ROS-mediated apoptosis [53]. Here, we provide evidence that activation of oxidative stress in response to heat stress results in the degradation of nuclear p-Akt, which correlates inversely with apoptosis and are essential in inhibiting apoptosis. There is evidence that direct interaction of PKM2 and Akt contributes to not only phosphorylate Akt, but also PKM2 itself, which is essential for the nuclear translocation of PKM2 protein and its induction for cyclin D1 expression [54]. Hsp90 can promote their interaction, just like our data showing that increased expression of Hsp90 induced by aspirin demonstrated a very prominent reversal effect for nuclear p-Akt reduction, resulting in further degradation of Akt protein and a partial decline in PKM2 expression explosion (potential increase in PKM2 phosphorylation). However, unlike p-Akt, nuclear HIF-1α, as another key antiapoptotic factor, presented a general induction situations in heat-stressed groups. A study on *Daphnia pulex* from Klumpen et al., turned out that, respond to peroxidation caused by heat stress, nuclear HIF-1α gave rise to peak at 1 h of heat stress, decreased to the level before heat stress at 2 h, then continued to reduce from 6 h [55]. Our data showed that the renal HIF-1α in HS was still induced at 5 h of heat stress, implying that the HIF-1α induction could be retained longer (more than 5 h) in mouse than *Daphnia pulex*. It could be predicted that the HIF-1α would decrease to the lower level with the increasing time of heat stress, just like a preliminary reduction in GA+HS and TR+HS (compared to those in GA and TR), in which groups oxidative stress was more serious (higher MDA levels) than other groups, and then resulted in more degradation of HIF-1α. Our data do not show strong/conclusive correlation between the apoptosis rates and the levels of nuclear PKM2 and Akt, implying that heat stress may be not strictly restricted to oxidative stress. In addition, heat stress and/or aspirin increased nuclear and cytoplasmic HSF-1 levels, which was responsible for the upregulation of Hsp90 and Hsp70.

Induction of heat stress-induced autophagy-mediated survival was further investigated. As known, the activation of mTOR can inhibit autophagy. Under the condition of heat stress, ASA upregulated the heat-stressed autophagy level and GA or TR restricted it, because p-mTOR is low in ASA+HS, and high in GA+HS or TR+HS. However, this pattern was contrary to the classical inhibition of Hsp90-Akt on mTOR signaling, suggesting that mTOR is regulated by other pathways under heat stress. Hypoxia is a condition in which tissues are starved of oxygen, is also observed in the heat-stressed kidney and is characterized by increased MDA. HIF-1α, a transcription factor, is a key factor in hypoxic environments, contributing to cell metabolism and survival [56,57,58]. Consistent with these reports, our study revealed marked HIF-1α accumulation in kidney tissue in response to heat stress. Increasing evidence has confirmed that HIF-1α plays an important role in autophagy progression by either repressing mTOR or enhancing BNIP3/BNIP3L, which disrupts the interaction between Beclin 1 and Bcl-2/Bcl-xL to recover the autophagic function of Beclin 1 [21,36]. Our data demonstrated that HIF-1α accumulation in the HS group enhanced BNIP3 and BNIP3L levels, which was probably responsible for heat stress-induced autophagy. In addition, Hsp90 interacts with HIF-1α and inhibits HIF-1α ubiquitination, ultimately leading to HIF-1α aggregation [56]. Coimmunoprecipitation showed that the interaction was strengthened by heat stress and further induced by increased Hsp90 expression resulting from aspirin treatment. Compared to the effects in the HS group, the stronger interaction of Hsp90 and HIF-1α in the ASA+HS group resulted in more HIF-1α accumulation, thus inducing more BNIP3 and BNIP3L. In detail, in this study, the autophagy levels in all heat-stressed groups were agreement with the pattern of BNIP3 expression, while BNIP3L expression in HS and ASA+HS was consistent with their autophagy levels, but not inconsistent with the autophagy in GA+HS and TR+HS. Bellot et al. think that their study clearly reveals that BNIP3 and BNIP3L can act separately, but the strength of the response can be enhanced when BNIP3 and BNIP3L act together [21]. These indicates that, during heat stress, BNIP3 plays an indispensable role in inducing autophagy, and BNIP3L exerts only a auxiliary role or serves as a facilitator in this process. Intriguingly, the phosphorylation of mTOR was also reversed by aspirin, suggesting that mTOR inactivation during heat stress was mediated by the Hsp90-HIF-1α axis. Further data indicated that as a transcriptional coactivator, nuclear HIF-1α was also increased. Nuclear HIF-1α binds to hypoxia response elements to reprogram cellular metabolism, ultimately increasing extra oxygen consumption and reducing glucose uptake and lactate production [59]. Aspirin intensifies the process of HIF-1α nuclear translocation through its interaction with Hsp90.

To confirm the above observations, the results in the GA and GA+HS groups were analyzed. GA significantly inhibited the chaperone function of Hsp90 with PKM2 and Akt, thus directly negatively influencing the renal levels of PKM2, Akt activation, and HSF-1. These aspects severely disrupted the survival ability of the heat-stressed kidney. Therefore, although GA treatment did not alter renal Hsp90 levels, pathological tissue damage, peroxidation, and cellular apoptosis were markedly enhanced. Previous studies have shown that Hsp90 promotes mitochondrial translocation of cytoplasmic Akt [60]. The present results showed that deficiencies in Hsp90 function were also unfavorable for the nuclear translocation of Akt, p-Akt and downstream HSF-1, excluding PKM2. The reduction in nuclear p-Akt negatively influences its retention and function in the nucleus and the ability to inhibit apoptosis [61,62]. The decreased interaction of Hsp90 with HIF-1α following GA treatment partially abrogated the restriction of mTOR phosphorylation and promotion of BNIP3 expression, contributing to the significant decrease in autophagy-mediated survival.

To determine which proteins activate Akt signaling, the data from the TR and TR+HS groups were investigated. As expected, despite having no influence on the interaction between Hsp90 and Akt, TR administration hindered the phosphorylation of Akt, including its nuclear translocation, resulting in the accumulation of intracellular Akt, especially during heat stress. Moreover, PKM2 synthesis was restricted due to the negative feedback of excessive Akt protein. Regarding downstream Akt activation, HSF-1 showed a significant decrease in protein quantity, and the remaining protein was still preferentially translocated into the nucleus. This is consistent with a previous study that showed a substantial downregulation of HSF-1 upon the inhibition of Akt activation [30]. In addition, the nuclear translocation of HSF-1 was instrumental in maintaining the expression of different heat shock proteins during physiological stress responses [63], as verified by the reduction in Hsp70 expression in the TR+HS group compared to that in the HS group. In the absence of p-Akt, p-mTOR increased, further indicating that the heat-stressed phosphorylation of mTOR was not controlled by Akt signaling. Furthermore, HIF-1α was downregulated with TR treatment, possibly because of the feedback regulation of high levels of p-mTOR, and the decreased BNIP3 levels during heat stress. The limited amount of HIF-1α was also preserved at the same level as that in the HS group. Taken together, our data demonstrated that HIF-1α but not the inhibition of Akt was a pivotal factor in heat stress-induced autophagy by altering the phosphorylation of mTOR and expression of BNIP3/BNIP3L. Akt signaling mainly functions in resisting apoptosis during heat stress.

## 4. Materials and Methods

### 4.1. Animals and Sampling

C57BL/6JNju male mice (6 weeks old, 20–25 g) were purchased from GemPharmatech Co., Ltd. (Nanjing, Jiangsu, China). First, 80 mice were divided randomly into eight groups: Control (Con), ASA, GA, TR, HS, ASA+HS, GA+HS, and TR+HS groups. The mice were housed conventionally for one week and then used to assess the effect of heat stress on renal tissues. The ASA and ASA+HS groups were administered aspirin (Sigma Chemical, St. Louis, MO, USA; dissolved in distilled water) by intragastric administration (1 mg/kg body weight) before 2 h of heat stress. The GA and GA+HS groups were treated with geldanamycin (Beyotime, Haimen, Jiangsu, China; dissolved in dimethyl sulfoxide (DMSO) and diluted with distilled water; DMSO concentration < 0.1%) by intraperitoneal injection according to the manufacturer’s instructions (50 mg/kg body weight) before 14 h of heat stress. The TR and TR+HS groups were treated with triciribine (Beyotime; dissolved in DMSO and diluted with distilled water; DMSO concentration < 0.1%) according to the manufacturer’s instructions (1 mg/kg body weight) before 14 h of heat stress. The Con and HS groups were treated with saline only. Then, the HS, ASA+HS, GA+HS, and ASA+HS groups were exposed to heat stress for 5 h by rapidly increasing the air temperature from 25 ± 1 to 42 ± 1 °C in a phytotron while maintaining the humidity at 75%. Meanwhile, the Con, ASA, GA, and TR groups were kept under normal conditions (25 ± 1 °C, humidity 75%) for the same amount of time. All mice were allowed free access to feed and water during the experiment.

After anesthesia with diethyl ether and orbital blood collection, the animals were sacrificed and sampled. For each animal, one kidney was placed in liquid nitrogen and stored at −80 °C for later homogenization and biochemical determinations, and the other kidney was fixed in 4% paraformaldehyde for morphological detection. All animal experiments were undertaken following the guidelines of the Regional Animal Ethics Committee and were approved by the Institutional Animal Care and Use Committee of Nanjing Agricultural University (identification code: PZ2018009, 5 November 2018).

### 4.2. Clinical Observation and Scoring

During the entire experiment, the clinical patterns (respiratory rate, food and water intake and behavior) of all mice were observed and scored according to the following standards: dead (4 points); heavily depressed mental state, curled up under cushion material, not drinking water, deep and fast breathing (3 points); moderate depression, increased drinking water, faster breathing (2 points); mild depression (1 point); or normal (0 points). The scores were compared among the groups.

### 4.3. Assessment of Kidney Damage

Mouse blood samples were centrifuged at 4000× *g* for 5 min to obtain the serum, which was sent to Super Biotech Co., Ltd. (Nanjing, Jiangsu, China) to detect the activities of BUN and AST. Kidney tissues of five animals per group were selected, and paraffin embedding and tissue processing were performed. Sections at a thickness of 3 μm were taken and stained with hematoxylin and eosin (H&E). Tissue damage was evaluated under a light microscope at 400× magnification by two pathologists who were blinded to the treatments. Damage was analyzed and scored as follows: no obvious pathological change (0 points), cellular swelling or mild degeneration (1 point), middle cellular degeneration (2 points), serious degeneration (3 points), and necrosis of epithelial cells in renal tubules (4 points).

### 4.4. Extraction of Total Protein and Determination of Oxidation and Antioxidant Markers

RIPA buffer supplemented with protease inhibitor cocktail (CWbio, Nanjing, Jiangsu, China) was added to the frozen tissue, which was homogenized and centrifuged at 14,000× *g* at 4 °C for 10 min to obtain the supernatant (total protein). Protein concentrations were quantified by the BCA method. Peroxidation of kidney tissues was estimated by measuring the MDA content, and antioxidant status was assessed by detecting the GSH-Px and CAT levels, all of which were conducted using the corresponding diagnostic kits (Jiancheng, Nanjing, Jiangsu, China) according to the manufacturer’s instructions.

### 4.5. Extraction of Nuclear and Cytoplasmic Proteins

Nuclear and cytoplasmic proteins were extracted using a nuclear and cytoplasmic extraction kit (CWbio) according to the manufacturer’s protocol. All protein concentrations were quantified with the BCA method.

### 4.6. Western Blotting

Equal amounts of extracted protein samples were mixed with 5× sodium dodecyl sulfate (SDS) sample buffer, boiled for 10 min, and then separated by SDS-polyacrylamide gel electrophoresis (PAGE). The proteins were transferred to nitrocellulose membranes after electrophoresis. The membranes were incubated with 5% skim milk, followed by primary antibodies overnight at 4 °C. The primary antibodies were obtained from Proteintech (Wuhan, Hubei, China; Hsp90) and Zen BioScience (Chengdu, Sichuan, China; Hsp70, PKM2, Akt, p-Akt, HSF-1, Caspase-3, AIF, PNCA, mTOR, p-mTOR, HIF-1α, and β-actin), Abbkine (Wuhan, Hubei, China; LC3-I/II, BNIP3 and BNIP3L). After washing with TBST, the membranes were incubated for 2 h with horseradish peroxidase (HRP)-conjugated secondary antibodies. Immunoreactive protein bands were visualized using an electrochemiluminescence substrate and a gel imaging system (Tanon Science and Technology, Shanghai, China). Finally, the reactive bands were quantified using Image J software (National Institutes of Health, New York, USA). β-actin or PNCA (proliferating cell nuclear antigen ) was used as the loading control.

### 4.7. Immunohistochemistry

The deparaffinized sections were rehydrated, followed by antigen retrieval. Tissue sections were successively blocked with 0.1% Triton X-100 and then with goat serum at room temperature. The slides were then incubated with primary antibodies against LC3-II at a dilution of 1:200 at 37 °C for 1 h. After washing in phosphate-buffered saline (PBS) with Tween-20, the slides were incubated with the corresponding HRP-labeled secondary antibody and then re-stained with hematoxylin. Images were acquired under a light microscope. At last, the positive signal areas (%) were quantified using Image J software.

### 4.8. Coimmunoprecipitation

For each sample of renal tissue lysates, 0.3 mL of lysate was incubated with 1 μg of Hsp90 antibody or control IgG (Santa Cruz, Dallas, TX, USA) overnight at 4 °C, followed by incubation with protein G-Sepharose beads (40 μL, Absin, Shanghai, China) for 2 h at 4 °C. The immune complexes were washed 3 times with RIPA buffer and then analyzed by western blotting with the indicated antibodies. The reactive bands were quantified using Image J software. Intensities of bands in Con were set as 1.

### 4.9. TdT-Mediated dUTP Nick End Labeling (TUNEL) Staining

Five-micrometer sections of kidney tissues embedded in paraffin were used. After deparaffinization in xylene, hydration with ethanol, and rinsing with distilled water, the sections were assayed using a TUNEL kit (KeyGEN, Nanjing, Jiangsu, China) according to the manufacturer’s protocol. The sections were counterstained with hematoxylin and observed under a light microscope, and apoptotic cells were counted in a blind manner to calculate the apoptosis rate.

### 4.10. Statistical Analysis

The data are expressed as the mean ± standard deviation. Differences among groups were tested by one-way analysis of variance (ANOVA) followed by Duncan’s multiple range test by using SPSS 25.0 version (IBM, Chicago, IL, USA). *p* < 0.05 was defined as statistically significant.

## 5. Conclusions

**Figure 7:**
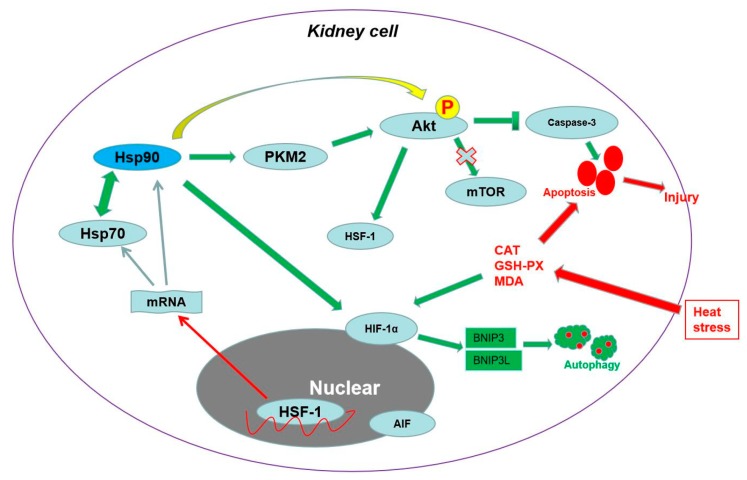
Schematic representation of Hsp90-mediated resistance to heat-stress damage in the kidney. Heat stress induced obvious renal histopathological and oxidative injury, which was connected with cellular apoptosis and autophagy. Under the assistance of Hsp70, Hsp90 played an important role in resisting apoptosis through the PKM2-Akt-HSF-1 axis and inducing autophagy-mediated survival through HIF-1α-BNIP3/BNIP3L signaling, ultimately protecting the kidney from heat stress-induced injury. In addition, the nuclear translocation of p-Akt and HSF-1 was instrumental in regulating the cellular ability of resisting heat-stress injury.

In summary, our study revealed a hitherto undescribed response to heat stress in the kidney in vivo. As shown in Figure 7, heat stress induced obvious renal histopathological and oxidative injury, which was connected with cellular apoptosis and autophagy. Hsp90, with the assistance of Hsp70, exerted an essential antiapoptotic effect through the PKM2-Akt-HSF-1 axis, which also induced Hsp70 expression. Meanwhile, Hsp90 regulated autophagy-mediated survival by the HIF-1α-BNIP3/BNIP3L pathway. Finally, both antiapoptosis and survival autophagy caused by Hsp90 protected the kidney from heat-stress injury. In addition, the nuclear translocation of p-Akt and HSF-1 could be also influenced by Hsp90 and played an important role in regulating the cellular ability of resisting heat-stress injury.
